# Dissecting Regulatory Networks of Filopodia Formation in a *Drosophila* Growth Cone Model

**DOI:** 10.1371/journal.pone.0018340

**Published:** 2011-03-28

**Authors:** Catarina Gonçalves-Pimentel, Rita Gombos, József Mihály, Natalia Sánchez-Soriano, Andreas Prokop

**Affiliations:** 1 Wellcome Trust Centre for Cell-Matrix Research, Faculty of Life Sciences, Manchester, United Kingdom; 2 Center for Neuroscience and Cell Biology, University of Coimbra, Coimbra, Portugal; 3 Biological Research Center, Hungarian Academy of Sciences, Institute of Genetics, Szeged, Hungary; University of Texas MD Anderson Cancer Center, United States of America

## Abstract

F-actin networks are important structural determinants of cell shape and morphogenesis. They are regulated through a number of actin-binding proteins. The function of many of these proteins is well understood, but very little is known about how they cooperate and integrate their activities in cellular contexts. Here, we have focussed on the cellular roles of actin regulators in controlling filopodial dynamics. Filopodia are needle-shaped, actin-driven cell protrusions with characteristic features that are well conserved amongst vertebrates and invertebrates. However, existing models of filopodia formation are still incomplete and controversial, pieced together from a wide range of different organisms and cell types. Therefore, we used embryonic *Drosophila* primary neurons as one consistent cellular model to study filopodia regulation. Our data for loss-of-function of capping proteins, enabled, different Arp2/3 complex components, the formin DAAM and profilin reveal characteristic changes in filopodia number and length, providing a promising starting point to study their functional relationships in the cellular context. Furthermore, the results are consistent with effects reported for the respective vertebrate homologues, demonstrating the conserved nature of our *Drosophila* model system. Using combinatorial genetics, we demonstrate that different classes of nucleators cooperate in filopodia formation. In the absence of Arp2/3 or DAAM filopodia numbers are reduced, in their combined absence filopodia are eliminated, and in genetic assays they display strong functional interactions with regard to filopodia formation. The two nucleators also genetically interact with enabled, but not with profilin. In contrast, enabled shows strong genetic interaction with profilin, although loss of profilin alone does not affect filopodia numbers. Our genetic data support a model in which Arp2/3 and DAAM cooperate in a common mechanism of filopodia formation that essentially depends on enabled, and is regulated through profilin activity at different steps.

## Introduction

F-actin networks are the structural determinants of cell shape and morphogenesis. They constitute the sub-membranous matrices of the cell cortex and of adhesion complexes, the lattice-like networks of lamellipodia and pseudopods/invadipodia, the bundles that form filopodia, spikes, stress fibres, microvilli or spines [1]. The actin regulatory machinery responsible for these sub-cellular arrangements comprises different classes of proteins, such as F-actin nucleators (e.g. Arp2/3, formins), filament bundlers (e.g. fascin), membrane deforming factors (e.g. BAR domain proteins), regulators of actin polymerisation (e.g. Ena/VASP proteins, profilin, capping proteins) or disassembly (e.g. ADF/cofilin), and actin-associated motors (e.g. myosin II, myosin X) [1], [2], [3]. For many of these proteins we have a good understanding of how they function biochemically. But how their activities integrate at the cellular level to orchestrate F-actin networks is little understood [2], [4]. For example, the formation of filopodia is being controversially discussed [5], [6], [7], [8], [9], [10]: the convergent elongation model proposes that Arp2/3-seeded actin filaments are promoted by factors such as Ena/VASP and fascin to elongate and assemble into filopodial bundles; in contrast, the *de novo* nucleation model proposes that formins assemble into sub-membranous complexes that nucleate parallel actin filaments *de novo* which then elongate into filopodial bundles. However, it remains unclear, whether these two putative modes of filopodia formation co-exist in the same cells, or might reflect cell-type or organism-specific mechanisms.

Various causes account for the poor understanding of actin network regulation at the cellular level. For example, the wealth of existing cellular data for actin regulators has been obtained from a wide range of different organisms and cell types. Therefore, any molecular models have to be pieced together on the premise that mechanisms are the same in different cellular contexts. Furthermore, to gain an understanding of how the various actin regulators functionally integrate, we need cellular systems that enable us to dissect complex genetic networks. The experimental repertoire provided by most current cellular systems still has limitations that slow down progress. As a promising strategy to overcome some of these problems, we have established a culture system for the study of axonal growth in embryonic primary neurons of *Drosophila* [11]. As typical of growing neurons, *Drosophila* primary neurons display prominent growth cones at the tips of their axons, which display highly dynamic motility needed to direct axon extension. Their motility is implemented by high F-actin content that drives the formation of prominent filopodia and lamellipodia [12]. We recently reported, that the filopodia of *Drosophila* growth cones perform protrusion, retraction, bifurcation, kinking, lateral drift and F-actin backflow, with characteristics and at rates very similar to those reported for neurons of mammals or other vertebrates [11]. Therefore, filopodia of *Drosophila* growth cones provide suitable readouts to study the functions of actin regulators [13], [14], and these regulators are evolutionarily well conserved [15].

Here we build on these possibilities and explore the regulatory networks that underlie filopodia formation, focussing on actin nucleators (Arp2/3, DAAM) and regulators of actin filament elongation (DAAM, CapA, CapB, Ena, profilin). Our loss-of-function studies of these proteins demonstrate characteristic roles in filopodia number and length, which are consistent with existing reports for their vertebrate homologues and demonstrate therefore the applicability of the *Drosophila* model. Data obtained from our genetic interaction studies support a model in which formins and Arp2/3 collaborate in one mode of filopodia formation, which largely depends on the function of enabled and is further facilitated by profilin. Remarkably, all these data were obtained in a uniform cellular model system, demonstrating its power to determine functional relationships across different classes of actin regulators in a cellular context.

## Results and Discussion

### Genetic support for the convergent elongation model

**Figure 1:**
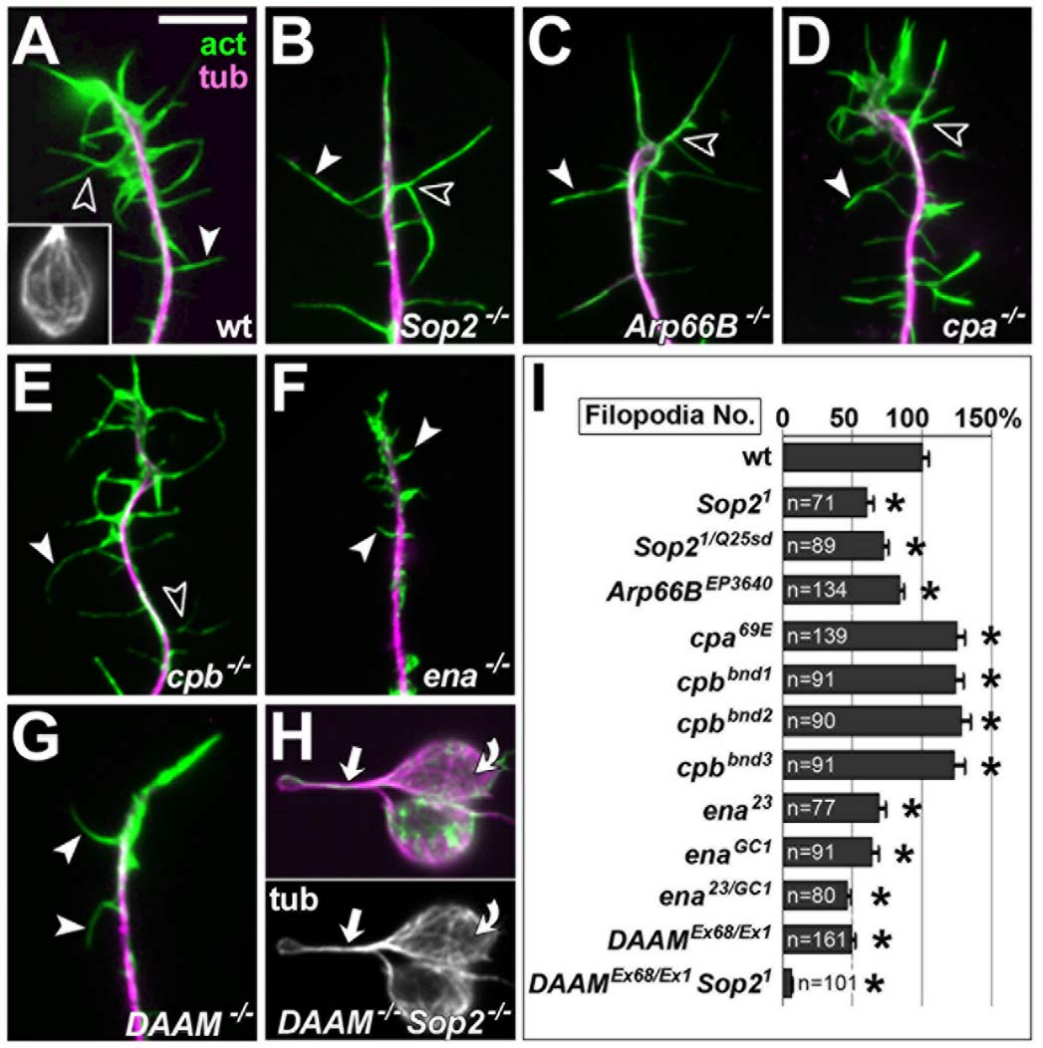
Filopodial phenotypes in primary neurons with loss-of-function of different actin regulators. **A–H)** Images of primary *Drosophila* neurons stained against actin (act; green) and tubulin (tub; magenta): wildtype control (A), *Sop2^1/Q25sd^* mutant (B), *Arp66B^EP3640^* mutant (C), *cpa^69E^* mutant (D), *cpb^bnd3^* mutant (E), *ena^23/GC1^* mutant (F), *DAAM^Ex68/Ex1^* mutant (G), *DAAM^Ex68/Ex1^ Sop2^1/Q25sd^* double mutant (H); white arrowheads point at examples of filopodia, open arrowheads at examples of bifurcating filopodia; greyscale images show tubulin staining in neurites (arrow in H) and cell bodies (curved arrow in H and inset in A). **I)** Filopodia numbers in neurons carrying different homozygous/heteroallelic combinations of mutant alleles of actin regulators (as indicated); sample numbers (n) and statistical significances are indicated (asterisks represent P≤0.005; Mann-Whitney rank sum test). Scale bar (in A) represents 4 µm in A–G and 10 µm in H and inset in A.

The convergent elongation model proposes Arp2/3 as the crucial nucleator [5], [6], [7]. To test whether Arp2/3 is required for filopodia formation, we cultured primary neurons derived from *Drosophila* embryos carrying loss-of-function mutations in the *Sop2* gene encoding the ArpC1/p40 subunit of the Arp2/3 complex, or a mutation in the *Arp66B* gene encoding the Arp3 subunit (*Sop2^1^*, *Sop2^Q25sd^*, *Arp66B^EP3640^*; all mutant alleles used in this study are well characterised, as detailed in Materials and Methods). Mutations in each of the three genes caused highly significant reductions in filopodia numbers (Fig. 1B, C, I). The degree of filopodial loss was comparable to knock-down studies in mouse neurons. Thus, the reduction, relative to wildtype, of filopodia numbers in *Sop2* loss-of-function mutant fly neurons (*Sop2^−/−^*) was comparable in strength to knock-down of the mouse p34 subunit (60–76% in fly *vs.* 70–73% in mouse); deficiency of Arp3 caused a slightly milder phenotype both in fly and mouse cells (84% *vs.*≈86%; Fig. 1I) [16].

Capping proteins are expected to act as negative regulators of the convergent elongation process, since they are potent inhibitors of barbed end-elongation of actin filaments [17] and negative regulators of nucleation processes [18]. We investigated neuronal cultures extracted from embryos carrying mutant alleles for either the capping protein α or the capping protein β (*cpa^69E^*, *cpb^bnd1^*, *cpb^bnd2^*, *cpb^bnd3^*). The *cpa^−/−^* or *cpb^−/−^* homozygous mutant neurons showed a consistent increase to about 125% in filopodia number (Fig. 1D, E, I). These data are in agreement with observations in migrating mammalian cells [19] and confirm a negative role of capping proteins in filopodia formation.

Ena/VASP is considered a key player in the convergent elongation process. Thus, it is an efficient anti-capping factor, a key promoter of actin polymerisation, and it can cluster the barbed ends of neighbouring actin filaments through its ability to oligomerise [20]. The *enabled* (*ena*) gene encodes the only *Drosophila* homologue of this family. Primary *Drosophila* neurons carrying well characterised *ena* loss-of-function mutant alleles (*ena^GC1^*, *ena^23^*) displayed severely reduced filopodia numbers (46–69%; Fig. 1F, I), as was similarly reported for epithelial cells at the leading edge during dorsal closure in *Drosophila* embryos [21], [22]. This finding is in agreement with loss-of-function analyses in mouse, *Dictyostelium* and *C. elegans*, all of which were reported to have an important but not an absolute requirement of Ena/VASP function for filopodia formation [23], [24], [25].

Taken together, our loss-of-function analyses of a number of actin regulators produced a set of data that is in line with existing reports for mammalian and other vertebrate and invertebrate cells, and is in principal agreement with the convergent elongation model of filopodia formation. Importantly, these data were all generated in the same cellular system, demonstrating its suitability for functional studies of actin regulator functions, and providing us with the unique opportunity to address their functional relationships directly in one consistent cellular context.

### Arp2/3 and formins are required for filopodia formation in the same cells

DAAM has been suggested to be the only formin in embryonic *Drosophila* neurons [15]. Accordingly, using the same *Drosophila* primary neuron system, we previously demonstrated a strong requirement of the formin DAAM for filopodia formation [14] (Fig. 1G, I). Therefore, both formins and Arp2/3 are important for filopodia formation in this system. To assess, whether this requirement coincides in the same cells, we tested combined loss-of-function of both nucleators. In cells carrying the strongest mutant alleles of *Sop2* and *DAAM* (*DAAM^−/−^ Sop2^−/−^* double mutant neurons), filopodia numbers were reduced to 5%, and weak phalloidin staining throughout these cells indicated very low F-actin content (Fig. 1H, I). In agreement with recent reports that filopodia serve as important facilitators of neurite initiation [23], we found that only 20% of *DAAM^−/−^ Sop2^−/−^* cells displayed neurites. In contrast, microtubule networks appeared unaffected in cell bodies of the double mutant neurons (Fig. 1A inset *versus* H), indicating that these cells were otherwise healthy.

We conclude that DAAM and Arp2/3 both contribute to filopodia formation in the same cells. The two together represent the key actin nucleators in *Drosophila* primary neurons, and any further potential nucleator activity appears insufficient to provide enough F-actin to induce filopodial protrusions. This finding provided a possibility to address the question of whether DAAM and Arp2/3 contribute to parallel populations of filopodia in the same cells through different mechanisms (convergent elongation *versus* de novo nucleation), or collaborate in a shared mechanism of filopodia formation.

### Arp2/3 and formins instate filopodia of similar appearance

**Figure 2:**
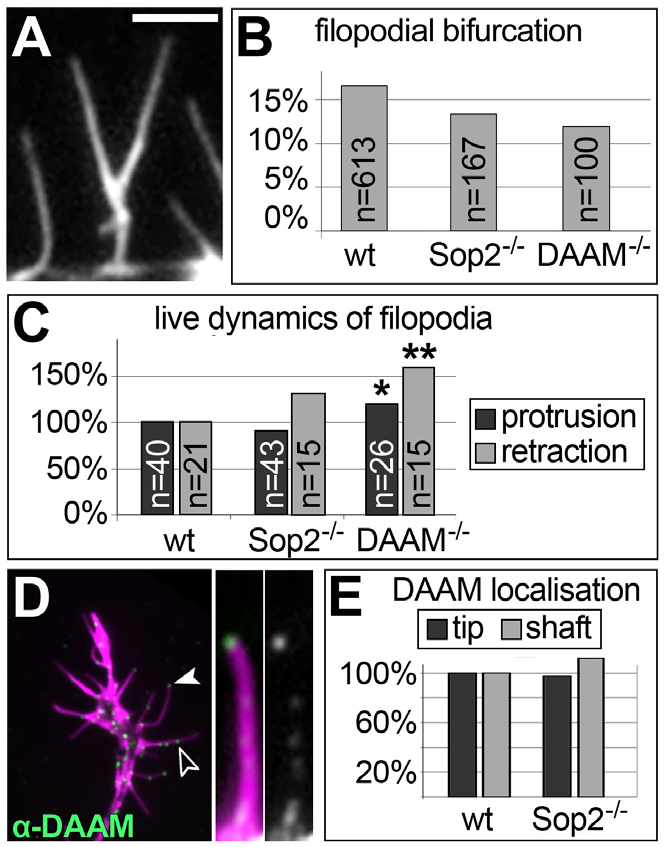
Loss of DAAM or Sop2 reveal similar morphologies. **A)** Bifurcated filopodium stained for actin. **B)** Quantification of relative numbers of bifurcated filopodia in wildtype controls, *DAAM^−/−^* and *Sop2^−/−^* mutant neurons (n, sample numbers). **C)** Quantification of protrusion and retraction rates of filopodia in live movies (*p = 0.051, **p = 0.006; Mann-Whitney rank sum test). **D)** Localisation of anti-DAAM at the tip (white arrow head) and along the shaft (open arrowhead) of filopodia in wild type growth cones; a magnified view of a filopodium is shown on the right (green channel shown in greyscale). **E)** Quantification of DAAM localisation in filopodia of wildtype neurons (as shown in D) and *Sop^−/−^* mutant neurons (not shown). Scale bar represents 5 µm in A and right side of D, 2.5 µm on the left side of D.

To assess their functional relationship, we first compared filopodia in *Sop2^−/−^* mutant neurons (displaying DAAM nucleator function) with those in *DAAM^−/−^* mutant neurons (displaying Arp2/3 nucleator function). We found that filopodia in *Sop2^−/−^* mutant and *DAAM^−/−^* mutant neurons were of similar shape, including occasional kinks and bifurcations (Fig. 1B, G); the frequency of bifurcations (which has previously been associated with the activity of formins) [26] was slightly reduced, but to similar degrees in both *Sop2^−/−^* and *DAAM^−/−^* mutant neurons when compared to wildtype (Fig. 2B). In live analyses, retraction and protrusion rates of filopodia were the same in *Sop2^−/−^* mutant neurons compared to wildtype, whereas *DAAM^−/−^* mutant neurons showed modestly increased protrusion rates and strongly increased retraction rates (Fig. 2C). This increase in protrusion and retraction rates is consistent with recently demonstrated polymerisation-enhancing and capping activities of DAAM at barbed ends of actin filaments [27]. Notably, DAAM is in the right position to influence filopodial length through such activities, since it localises to shaft and tips of filopodia in both wildtype and *Sop2^−/−^* mutant neurons (Fig. 2D, E) [14]. Therefore, changes in filopodia dynamics observed in *DAAM^−/−^* mutant neurons could be due to the fact that processive elongation in its absence is executed exclusively by other factors, in particular Ena [2].

Taken together, the only difference we found between Arp2/3- and DAAM-dependent filopodia regards the retraction and protrusion rates of filopodia. This difference is likely to relate to a function of DAAM in regulating actin polymerisation rather than nucleation, and is therefore distinct from its role in filopodia formation. Other aspects of filopodia appeared the same, irrespective of whether actin filaments are seeded by only Arp2/3, only formins or by both nucleators.

### Sop2 and DAAM act in the same regulatory network

**Figure 3:**
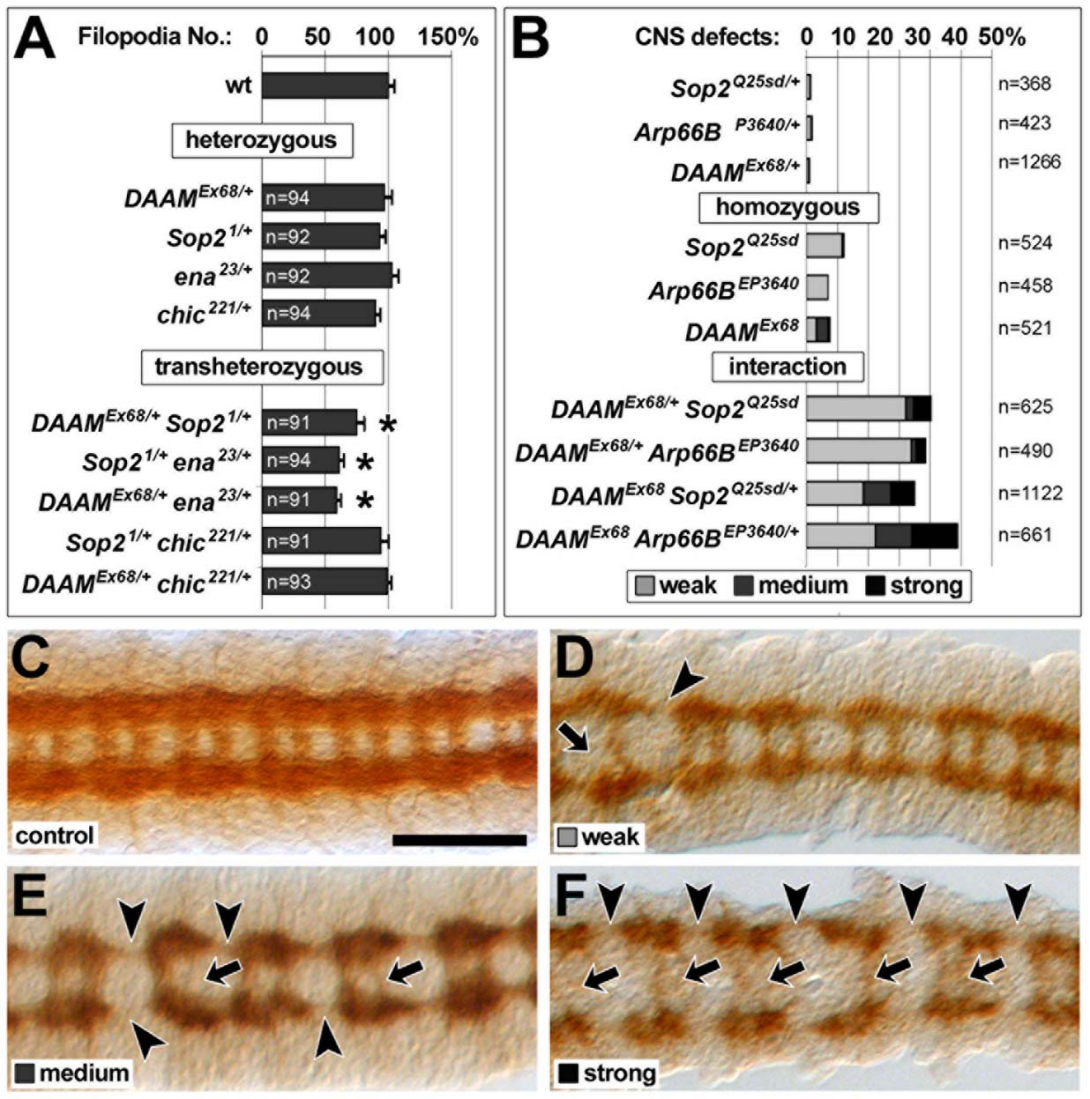
DAAM and Sop2 act in the same genetic networks. **A)** Filopodia numbers in neurons carrying different heterozygous or transheterozygous combinations of mutant alleles of actin regulators (as indicated); sample numbers (n) and statistical significances are indicated (asterisks represent P≤0.005; Mann-Whitney rank sum test). **B)** Quantification of CNS defects as described previously [14]. **C–F)** Representative images of ventral nerve cords at embryonic stage 16 stained with BP102 antiserum labelling the axonal compartments [49]; wildtype controls are shown in C and mutant embryos in D to F; breaks in commissures (arrows) or connectives (arrow heads) are classified with respect to their frequency into weak (breaks in 1–2 segments), medium (breaks in 3–5 segments) and strong (breaks in 6–10 segments) phenotypes (as quantified in panel B). Scale bar (in C) represents 50 µm.

To further assess their functional relationships, we carried out genetic interaction studies between *Sop2* and *DAAM*. Heterozygous mutant neurons carrying one mutant and one wildtype copy of either of the two genes (*Sop2^−/+^* or *DAAM^−/+^*), displayed no changes in filopodia numbers compared to wildtype (Fig. 3A). Therefore, reducing the abundance of either of the two nucleators was not rate limiting for filopodia formation. When one mutant copy was present for both genes simultaneously in the same neurons (transheterozygous condition; *DAAM^−/+^ Sop2^−/+^*), this combined reduction of both proteins became rate-limiting, and neurons displayed significantly reduced filopodia numbers (75%; Fig. 3A). This genetic interaction was confirmed by analyses in embryos, using structural aberrations in the CNS as well established readouts [14]. Thus, nervous system defects were low in embryos carrying mutant alleles of *DAAM*, *Sop2* or *Arp66B* alone, but were strongly increased when combining their mutant alleles (*DAAM^−/+^ Sop2^−/−^*; *DAAM^−/+^ Arp66B^−/−^*; *DAAM^−/−^ Sop2^−/+^*; *DAAM^−/−^ Arp66B^−/+^*; Fig. 3B). The dominant nature of these genetic interactions is an important indicator that the functions of both genes are likely to converge in the same molecular process.

We next took advantage of our observation that filopodial numbers are reduced in *ena^−/−^* mutant neurons and assessed potential genetic interactions of *ena* with *Sop2* and *DAAM*. Heterozygous *ena^−/+^* mutant neurons displayed normal filopodia numbers (Fig. 3A). However, if one mutant copy of *ena* was combined with one mutant copy of *DAAM* (*DAAM^−/+^ ena^−/+^*) or of *Sop2* (*Sop2^−/+^ ena^−/+^*), filopodia numbers were reduced to about 60% (Fig. 3A). This reduction was comparable in strength to values observed in neurons deficient for only Ena (46–69%), *Sop2* (60%) or DAAM (49%; Fig. 1I). We conclude that Ena is likely to functionally converge with the two nucleators in filopodia formation.

Therefore, like our morphological analyses, also the genetic interaction studies fail to provide any indications that the two nucleators act through distinct molecular machineries of filopodia formation.

### Profilin and Ena are required for filopodia elongation

Profilin acts as a powerful promoter of actin polymerisation *in vitro* and in cells; it is known to bind and functionally interact with Ena/VASP, DAAM and other formins, both in vertebrates and *Drosophila* [14], [20], [21], [27], [28], [29], [30], [31], [32]. However, little is known about the functional roles of profilin during filopodial formation.

**Figure 4:**
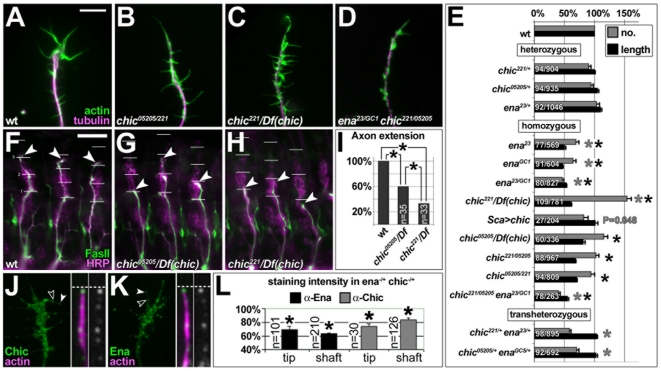
Profilin and Enabled regulate filopodial length and number. **A–D)** Images of primary *Drosophila* neurons stained against actin (act) and tubulin (tub); genotype as indicated. **E)** Mean filopodia numbers per neuron (grey) and mean filopodial length (black) of neurons carrying different heterozygous, homozygous/heteroallelic or transheterozygous combinations of mutant alleles of *ena* and/or *chic*, or with targeted expression of *UAS-chic* via *Sca-Gal4* (*sca>chic*); numbers before and after slash indicate sample size for filopodia number/length; grey/black asterisks indicate significance P≤0.001 for filopodia number/length (Mann-Whitney rank sum test). **F–H)** Embryos at stage 16 stained with anti-HRP (magenta) and anti-FasII (green; anterior to the left, dorsal at the top; genotype as indicated); three hemisegments are shown, respectively; white lines indicate dorsoventral scale relative to HRP landmarks [56]; arrowheads indicate tips of intersegmental motornerves. **I)** Quantification of motoraxonal extensions: 33% in *chic^221^*/Df(chic) and 60% in *chic^05205^*/Df(chic); asterisks like in E; n, number of assessed hemisegments. **J,K)** Localisation of anti-Chic and anti-Ena at the tip (white arrow head) and along the shaft (open arrowhead) of filopodia in growth cones of wildtype neurons (green channel shown in greyscale), as similarly observed for the Ena homologue Mena in mouse growth cones [35]. **L)** Quantification of staining intensities of Ena and Chic dots in filopodia of *ena^23/+^ chic^221/+^* transheterozygous mutant neurons (asterisks like in E). Scale bar (in A) represents 4 µm in A–D, 10 µm in F–H, 3 µm on the left and 1 µm on the right side of J, K.

The only profilin encoded by the *Drosophila* genome is called Chickadee (Chic). In neurons carrying the well characterised loss-of-function mutant alleles *chic^221^* and *chic^05205^*, filopodia lengths were reduced to 71–77% relative to wildtype (Fig. 4B, C, E). This shortening might be partly due to profilin's role in facilitating the activity of formins in actin polymerisation [2], [27]. An alternative explanation is the close cooperation of profilin with Ena/VASP in actin polymerisation [20], [33]. Accordingly, we found that both Ena and Chic localise to filopodia (Figs. 4J, K and S1). Furthermore, we found that, like *chic^−/−^*, also *ena^−/−^* mutant neurons display a reduction in filopodia length (47–53%; Figs. 1F and 4, E), and this is in agreement with reports for loss of Ena/VASP function in vertebrate neurons [34]. The degree of shortening found in *ena^23/GC1^*mutant neurons is not further enhanced in *chic^221/05205^ ena^23/GC1^* double-mutant neurons (53% *versus* 55%; Fig. 4D, E), consistent with a model in which both factors work in the same pathway. This view matches with the reported high affinity of Ena/VASP for profilin:G-actin in the context of actin polymerisation [20], [33]. From such a high affine interaction one would predict that protein levels have to be drastically reduced before any genetic interaction of *ena^−/+^* with *chic^−/+^* is revealed. In agreement with this prediction, we found that transheterozygous mutant neurons (*ena^−/+^ chic^−/+^*), which showed modest, though significant reductions in Chic and Ena levels (Fig. 4L), failed to display any filopodial length phenotypes (Fig. 4E).

### Profilin plays different roles in filopodia formation

Filopodia numbers were normal in *chic^05205/221^* or *chic^05205^*/*Df(chic)* mutant neurons, but they were increased to 154% in neurons carrying the *chic^221^* allele over a deficiency uncovering the *chic* locus [*chic^221^*/*Df(chic)*; Fig. 4B, C, E]. Although *chic^05205^* and *chic^221^* are well established strong loss-of-function mutant alleles, only *chic^221^* is a molecularly confirmed null allele (Materials and Methods). Therefore, we compared both alleles by quantifying motoraxonal stall phenotypes in *chic^−/−^* mutant embryos (Material and Methods). We found that *chic^05205^* caused significantly weaker axon stall phenotypes than *chic^221^* (33% extension in *chic^221/Df^* *versus* 60% in *chic^05205/Df^*; Fig. 4F–I). We conclude that the increase in filopodia numbers in *chic^221^* mutant neurons is likely to reflect the true amorphic (null mutant) condition. This interpretation is further supported by our finding that targeted over-expression of Chic in wildtype neurons caused a modest reduction in filopodia number (*sca>chic* in Fig. 4E). A potential molecular explanation for this negative role in filopodia formation is the reported inhibitory effect that profilins (and capping proteins) exert on actin nucleation *in vitro* [18], for example by competing for G-actin. In agreement with such opposing roles in nucleation, no genetic interactions of *chic* were found in transheterozygous constellations with *DAAM* (*DAAM^−/+^ chic^−/+^*) or *Sop2* (*Sop2^−/+^ chic^−/+^*; Fig. 3A).

In contrast, *chic* displayed a strong genetic interaction with *ena* in the context of filopodia formation: filopodia numbers were severely reduced in neurons which simultaneously carried one mutant allele of both genes, and this finding was confirmed using two independent allelic combinations (58% in *ena^23/+^ chic^221/+^*, 64% in *ena^GC5/+^ chic^05205/+^*; Fig. 4E). The reduction in filopodia numbers observed in *ena^23/GC1^* single-mutant neurons was not further enhanced in *chic^221/05205^ ena^23/GC1^* double-mutant neurons (46% *versus* 40%, not significant; Figs. 1F and 4D, E). These data suggest that profilin plays a second, positive role in filopodia formation which closely relates to the function of Ena/VASP. Ena clearly is the more important factor, directly executing anti-capping and clustering of barbed actin filament ends. Profilin is not required for anti-capping activities of Ena, but it can stimulate them [20]. Accordingly, filopodia numbers are reduced in *ena^−/−^* but not in *chic^−/−^* mutant neurons. Only when Ena levels are reduced (*ena^−/+^*), does additional reduction of profilin (*chic^−/+^*) become rate-limiting, thus explaining the reduction in filopodia numbers in *ena^−/+^ chic^−/+^* neurons. The genetic interaction observed here in the context of filopodia formation is consistent with genetic interactions observed in *Mena^−/−^ profilin-1^−/−^* mutant mice which were reported to display defects in neural tube closure [35].

Notably, *in vivo* analyses of *chic^05205^* and *chic^221^* mutant neurons produced contradictory results (abundant filopodia in embryonic motoraxons, lack of filopodia in pupal mushroom body neurons) [36], [37]. These findings might indicate that the different aspects of profilin function during filopodia regulation can be influenced through the different signalling events that orchestrate growth cone behaviours in time and space.

### Conclusions and perspectives

**Figure 5:**
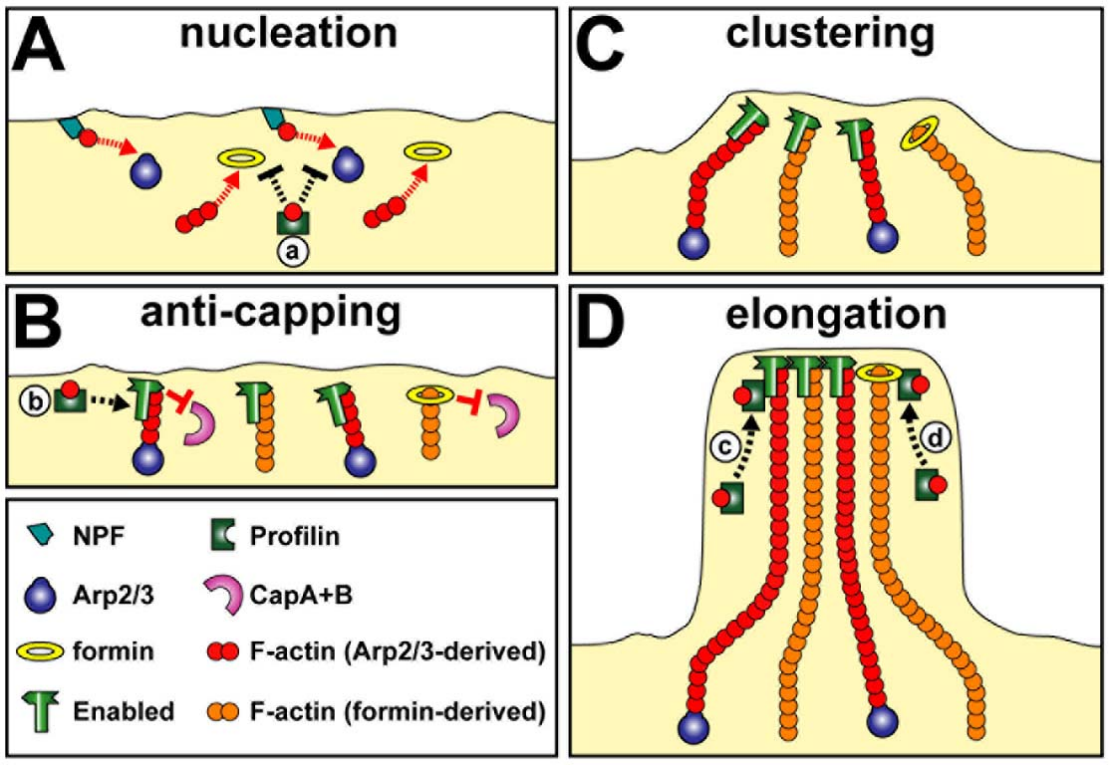
Model of filopodia formation consistent with known molecular interactions and functions, and the genetic data obtained in *Drosophila* neurons. **A)** Arp2/3 and the formin DAAM are the essential nucleators in *Drosophila* neurons; Arp2/3 is expected to require nucleation promoting factors (NPF), such as Scar [57]; in agreement with *in vitro* data [18], nucleation is negatively regulated by profilin (a; for example by competing for G-actin). **B)** Once nucleation occurred, barbed end polymerization becomes energetically favourable and can be promoted by DAAM [27]; inhibition of actin filament elongation through capping proteins is antagonised by formins and Ena [20], [31], [33]; anti-capping activities of Ena do not require profilin but can be stimulated by it (b) [20]. **C)** Through its tetramerising activity, Ena clusters the barbed ends of elongating actin filaments [20]; also DAAM might contribute to this clustering event, since it has F-actin bundling activity [27] and can bind Ena [14]. **D)** Processive actin elongation in filopodia of *Drosophila* growth cones is performed by DAAM and Ena; profilin potentially cooperates with both proteins in this context [20], [27], [31], [33] (c, d), but its cooperation with Ena appears more important for filopodial length regulation in cultured fly neurons.

By combining the power of *Drosophila* genetics with microscopic readouts for primary neurons, we were able to directly demonstrate functional relationships between different regulators of actin nucleation and polymerisation in filopodia formation. Importantly, all data generated here, were obtained in *Drosophila* primary neurons, i.e. one single cellular and experimental platform. We consistently found that loss of function of orthologous *Drosophila* and vertebrate actin regulators cause the same qualitative phenotypes; this finding adds to former reports that *Drosophila* and mouse spectraplakins have homologous functions in neuronal filopodia formation [13], and that the principal structure and dynamics of filopodia are well conserved [11]. We conclude therefore that work in *Drosophila* primary neurons provides a valid, efficient and promising strategy to advance our principal understanding of actin network regulation in higher eukaryotes. With respect to filopodia formation, our results do not support the existence of distinct modes of filopodia formation, but are consistent with a model in which formins and Arp2/3 cooperate in one common mechanism of filopodia formation. This view is supported by findings that formins can contribute to actin nucleation in lamellipodia of non-neuronal cells [38]. Therefore, we believe it to be more likely that the nucleating functions of formins and Arp2/3 contribute to a mixed pool of actin filaments, which serve as a substrate for convergent elongation processes of filopodia formation - essentially mediated by Ena (Fig. 5). Loss of either nucleator reduces the F-actin pool and hence limits the substrate required for filopodia forming processes, leading to less filopodia. Profilin influences these processes at different steps (Fig. 5). The generation of further mutant combinations and the analysis of further actin regulators (such as myosins, Bar-domain proteins or bundling factors) can now be used to validate, refine and extend this model.

## Materials and Methods

### Fly strains

All mutant alleles used in this study are well characterised. The following embryonic lethal, loss-of-function mutant alleles were used: ***ena^23^*** (from B. Baum) is caused by a nucleotide exchange introducing a STOP codon leading to a 52aa C-terminal truncation that deletes the EVH2 domain required for tetramerisation of Ena [39]. Furthermore, *ena^23^* displays an amino acid exchange (N379F) in the proline-rich domain with no known functional implications [39]. In *ena^23^* mutant background, anti-Ena staining (clone 5G2, mouse) is strongly reduced in primary neurons, CNSs and tendon cells (Fig. S1, A–D) [11], [40]. ***ena^GC1^*** (from Bloomington, stock #8569) is a protein null allele due to a chromosomal inversion (breakpoints at 55B and 56B5) which causes severe axonal growth phenotypes [41]; it is embryonic lethal over *ena^23^* [32] (own observations). ***ena^GC5^*** (from Bloomington, stock #8570) is caused by an inversion (breakpoints at 44E and 56B) [41]; it is embryonic lethal over *ena^GC1^* [39] (own observations). ***chic^221^*** (from D. van Vactor) is caused by an intragenic deletion in the *chic* gene removing 5′ non-coding and some of coding region of *chic;* it affects only the *chickadee* gene and is an obligate null allele [42] and amorph [36]; anti-Chic staining (mouse, clone chi1J) is strongly reduced in *chic^221^* mutant CNS and primary neurons (Fig. S1, I, J). ***chic^05205^*** (from D. van Vactor) is caused by a P-element insertion immediately upstream of the second coding exon [36] (FlyBase); anti-Chic staining is strongly reduced in *chic^05205^* mutant CNS and primary neurons (Fig. S1, G, H). ***Df(chic)*** (synonymous to *Df(2)GpdhA*; breakpoints at 25D7;26A8-26A9; from D. van Vactor) uncovers the *chic* locus [36]. ***Uas-eGFP-chic^13.2^*** is a kind gift from U. Thomas (unpublished). ***cpa^69E^*** (from F. Janody) is a null allele caused by a nonsense mutation at aa180 truncating the protein before its actin binding domain [43]. ***cpb^bnd1^*** (from F. Janody) is a C-to-T substitution causing a premature STOP codon at nucleotide 5 of the coding sequence [44]. ***cpb^bnd2^*** (from F. Janody) is a G-to-A substitution causing an E218K conversion [44]. ***cpb^bnd3^*** (from F. Janody) is a G-to-A substitution causing a E221K conversion [44]. ***Sop2^1^*** ( =  *ArpC1^CH60^*; from B. Baum) is caused by a 207bp genomic deletion that removes the last 62 codons of *Sop2* [45]. ***Sop2^Q25sd^*** (from Bloomigton, stock #9137) is caused by a point mutation in the conserved splice donor dinucleotide after Gln25 (C/gt→C/at) predicted to truncate the protein; it behaves as a null and is lethal over *Sop2^1^* [45]. **A** ***rp66B^EP3640^*** (from Bloomigton, stock #17149) is caused by a P-element insertion 138bp upstream of the predicted start codon; its lethality could be rescued by P-element excision [45]. Note that Arp2/3 complexes lacking Arp3 or Arpc1 have little or no nucleation activity [46], supporting the notion that mutations in these subunits abolish Arp2/3 activity [45]. ***DAAM^Ex1^*** is a hypomorphic, viable allele generated through imprecise excision of the *P{EP}EP1542* transposable element, resulting in deletion of most of the 3′UTR and a very small part of the C-terminal end of the coding region [47]. ***DAAM^Ex68^*** is a null allele generated through imprecise excision of the *P{EP}EP1542* element, resulting in deletion of the C-terminal 457 amino acids, including sequences corresponding to the ‘DAD’ domain and most of the ‘FH2’ domain [47]. ***DAAM^Ex1/Ex68^*** mutant neurons were harvested from embryos derived from homozygous *DAAM^Ex1^* mutant mothers crossed to *DAAM^Ex68^*, *Ubi::GFP/Y^Dp(1;Y)Sz280^* or *DAAM^Ex68^*, *arm-LacZ/Y^Dp(1;Y)Sz280^* males; this constellation is the strongest reported loss of *DAAM* function condition [14].

### Generation of primary cell cultures

The generation of primary cell cultures was carried out as described in detail elsewhere [11], [48]. In brief, cells were collected with micromanipulator-attached needles from stage 11 wildtype or mutant embryos (6–7 h after egg lay at 25°C) [49], treated for 5 minutes at 37°C with dispersion medium, washed and dissolved in the final volume of Schneider's medium [50] (Invitrogen; 5–6 µl/donor embryo), transferred to cover slips, kept as hanging drop cultures in air-tight special culture chambers [51] usually for 6 hr at 26°C.

### Stainings and documentation

Antibody stainings of primary neurons and embryos were carried out following standard procedures detailed elsewhere [52], [53], [54]. The following antibodies were used: anti-*Drosophila* Enabled (clone 5G2 raised against aa105-370 of Ena, mouse, 1∶20, DSHB, University of Iowa, IA, USA; for validation see Fig. S1) [55]; anti-Chickadee (clone chi1J, mouse, 1∶10, DSHB, University of Iowa, IA, USA; for validation see Fig. S1); anti-tubulin (clone DM1A, mouse, 1∶1000, Sigma; alternatively, clone YL1/2, rat, 1∶500, Chemicon); anti-*Drosophila* DAAM (rabbit, 1∶3000; published and validated elsewhere) [47]; anti-βGal (mouse, 1∶500, Promega Z3781); anti-FasII (clone ID4, mouse, 1∶20, DSHB); anti-GFP (goat, 1∶500, Abcam); Cy3 conjugated anti-HRP (goat, 1∶100, Jackson Immuno Research); FITC-, Cy3- or Cy5-conjugated secondary antibodies (donkey, purified, 1∶100–200; Jackson ImmunoResearch). Filamentous actin was stained with TRITC- and FITC-conjugated phalloidin (Sigma). Stained specimens were mounted in Vecta-shield mounting medium (Vector Labs). Standard documentation was carried out with AxioCam monochrome digital cameras (Carl Zeiss Ltd.) mounted on BX50WI or BX51 Olympus compound fluorescent microscopes. Live imaging was carried out on a Delta Vision RT (Applied Precision) restoration microscope using a [*60x/1.42 Plan Apo*] objective and the [*Sedat*] filter set (Chroma [*89000*]). The images were collected using a Coolsnap HQ camera (Photometrics).

### Quantifications and statistic analyses

Filopodia were identified as needle-like, phalloidin-stained surface protrusions; filopodia numbers reflect the total amount of filopodia per neuron; length was measured via ImageJ from the tip to the point at their base where filopodia dilate; protein levels were measured in ImageJ and represent the mean grey values at sites of protein accumulations. Quantification of motoraxonal growth and of CNS defects was performed as described elsewhere [14], [56]. Statistical analyses were carried out with Sigma Stat software using Mann–Whitney rank sum tests.

## Supporting Information

Figure S1 **Specificity of anti-Ena and anti-Chic antisera.** Images show horizontal views of late embryonic CNSs (A, C, E, G, I; anterior to the left) and growth cones of primary neurons (all other images); CNSs and neurons were derived from wildtype (wt) or mutant embryos (as indicated on top) and were stained with anti-Chickadee and anti-Enabled antisera (indicated on the left). With both antisera, mutant alleles of the respective gene caused a strong reduction in protein levels. Scale bar (in A) corresponds to 10 µm in A, C, E–I and 7 µm in B, D, F–J.
(TIF)
